# Hydration Kinetics of Composite Cementitious Materials Containing Copper Tailing Powder and Graphene Oxide

**DOI:** 10.3390/ma11122499

**Published:** 2018-12-08

**Authors:** Shuhua Liu, Qiaoling Li, Xinyi Zhao

**Affiliations:** State Key Laboratory of Water Resources and Hydropower Engineering Science, Wuhan University, Wuhan 430072, China; qllee@whu.edu.cn (Q.L.); xinyizhao@whu.edu.cn (X.Z.)

**Keywords:** composite cementitious materials, copper tailing powder, graphene oxide, hydration kinetics

## Abstract

The hydration heat evolution curves of composite cementitious materials containing copper tailing powder (CT) and graphene oxide (GO) with different contents are measured and analyzed in this paper. The hydration rate and total hydration heat of the composite cementitious materials decrease with the increase of CT dosage, but improve with the increase of CT fineness and GO dosage. The hydration process of the cementitious systems undergoes three periods, namely nucleation and crystal growth (NG), phase boundary reaction (I), and diffusion (D), which can be simulated well using the Krstulovic–Dabic model. The hydration rates of the three controlling processes of the composite cementitious system decrease with the increase of CT content, but improve slightly with the increase of CT fineness. GO enhances the controlling effect of the NG process of the cementitious systems with or without CT, thus promotes the early hydration as a whole.

## 1. Introduction

Copper tailings are waste materials generated during the purification of precious copper from the copper ores, and 128 t copper tailings will be left over per 1 t refined copper [1,2]. According to the United States Geological Survey Bureau, two trillion tons of copper tailings were produced worldwide in 2011 [3]. The cumulative copper tailings in China exceeded 3 trillion tons by 2014; however, only about 8.2% are recycled [3,4,5]. The rest are mainly disposed of in the tailing pond, which results in a lot of adverse effects. For example, the heavy metal elements in copper tailings poison the surrounding soil and water. Furthermore, as copper tailings are definitely solid gravel, the tailing pond is prone to destabilize and collapse in the case of earthquakes and flooding [6]. In addition, the construction cost and operation expense of a tailings depot are very high. As a result, the utilization of copper tailings in concrete as either fine aggregate or supplementary cementitious material is not only conducive to the resources recovery and utilization but also greatly reduces the environmental pollution, geological disasters, and other problems caused by copper tailings.

Some studies regarding the use of copper tailings as cement substitution have been conducted, and the results show that the pozzolanic activity of copper tailing powder is fairly low and its optimal replacement ratio is 5% without strength and durability reduction; however, the replacement ratio can reach up to 30–50% in mass concrete with consideration of the temperature control [2,7,8]. Moreover, it has been reported that graphene oxide (GO) has reinforcing and toughening effects on the cement paste [9,10,11,12,13]. The improvement of the mechanism of GO can be concluded using the following reasons. The first is called the template effect, in which GO can regulate the morphology of the hydration products of cement to form flower-shaped microcrystals with uniform shape and uniform distribution [9,10,11]. The second is the nucleation effect that GO lamellae can provide nucleation sites for nucleation and crystallization of calcium silicate hydrates (C-S-H), which promotes the crystallization, nucleation, and growth of C-S-H, thereby accelerating the early hydration [12]. In addition, the interface enhancement effect between GO and C-S-H is proven to be fairly high, which may also contribute to the strength and toughness of the paste [13].

As a result, copper tailing powder (CT) shows some adverse effects on early hydration and strength of the cement-based materials, but GO can improve their microstructure and properties. What will happen when the positive and the negative elements interact? How does GO promote the hydration mechanism of a cement-CT composite system? We suppose that GO also has reinforcing and toughening effects on the composite cementitious materials containing CT. In order to investigate the effect of CT on the early hydration mechanism of cement, as well as the enhancing effect of GO on the hydration of cement and cement-CT binder, the hydration heat evolution rate and cumulative hydration heat of composite cementitious materials containing CT and GO at different contents are measured at 25 °C by an isothermal calorimeter. Based on the thermodynamic data, the hydration kinetics of the composite cementitious materials containing CT and GO are investigated in detail using the Krstulovic–Dabic model to reveal the effects of CT and GO on the early hydration.

## 2. Materials and Methods

### 2.1. Raw Materials

**Figure 1:**
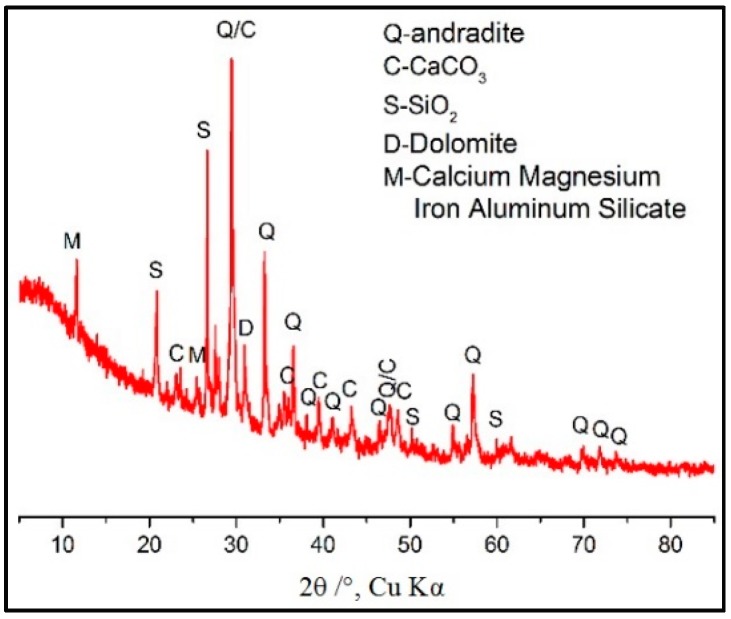
XRD pattern of CT.

**Figure 2:**
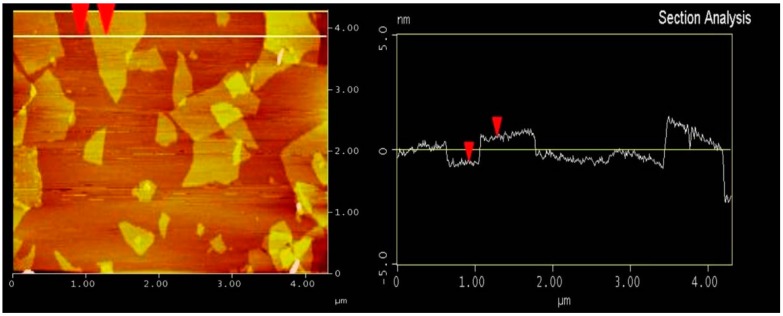
AFM image of graphene oxide (GO).

Ordinary Portland cement (P.O 42.5) and CT supplied by China Construction Mining Corporation are used in the experiment. Their chemical compositions, determined by the X-ray fluorescence (XRF Axios FAST, Malvern Panalytical Ltd., Royston, UK), are listed in Table 1. CT contains a great deal of CaO, SiO_2_, and Al_2_O_3_. While the total content of SiO_2_, Al_2_O_3_, and Fe_2_O_3_ is 53.03%, which is less than 70% of that put forward by ASTM C618-15 Standard Specification for Coal Fly Ash and Raw or Calcined Pozzolan for use in concrete [14]. Its XRD pattern is displayed in Figure 1; the XRD patters shows that SiO_2_ exists mainly in the form of andradite instead of active SiO_2_. In general, the CT used in this study has a low pozzolanic activity. GO dispersion is produced by Shanxi Institute of Coal Chemistry, Chinese Academy of Sciences, Taiyuan, China. It has a GO content of 4 mg/mL and its morphology, size (about 1 μm) and thickness (about 1 nm) are characterized by the Atomic Force Microscope (AFM Tosca™ 400, Anton Paar Shanghai Trading Co. Ltd., Shanghai, China) as shown in Figure 2.

### 2.2. Testing Methods

**Figure 3:**
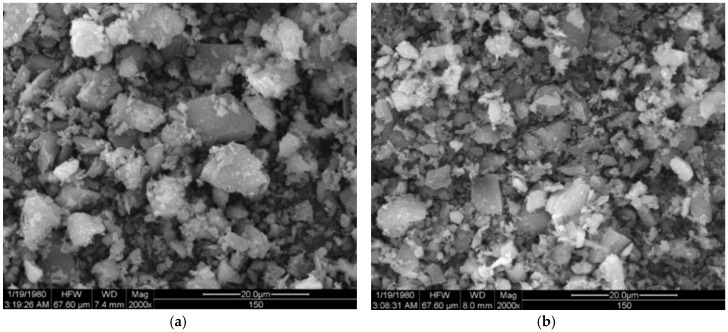
Particle morphology of CT after being ground for (**a**) 30 min; and (**b**) 60 min.

**Figure 4:**
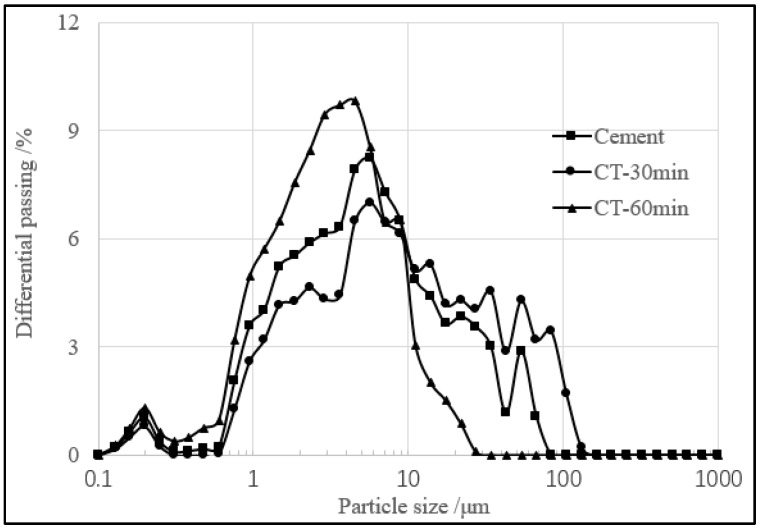
Particle size distribution curves of cement and CT.

The copper tailings were dried, first, at 60 °C and filtered through a 1 mm-square-hole sieve in order. Then, they were ground for 30 min and 60 min, respectively, in a small insulative ball mill (SM 500, Daoxu Machinery Factory, Shangyu, China). The ball mill has a speed of 48 r/min, with a loading capacity of 5 kg of sample. The powder morphology was investigated using scanning electron microscopy (JSM-5610LV, JSM Ltd., Tokyo, Japan). As shown in Figure 3, CT particles ground for 30 min and 60 min show irregular blocky, granular, and clastic shape. The particle size distribution of cement and CT, as shown in Figure 4, was measured by a laser particle size analyzer (Master size 2000, Malvern Instruments Ltd., Worcestershire, UK), ranging from 0.1 to 1000 μm. The specific surface area was calculated by software included in the laser particle size analyzer. The specific surface area of CT ground for 30 min and 60 min is 380 m^2^/kg and 690 m^2^/kg, respectively. The specific surface area of cement is 440 m^2^/kg. The average size of CT ground for 30 min and 60 min is 6.79 μm and 3.47μm, respectively, and the average size of cement is 5.67 μm, which is similar to their specific surface area.

Two paste systems, I and II, prepared for hydration heat determination are listed in Table 2 and Table 3, respectively. The hydration heat evolution rate and total hydration heat emission of the samples were measured by an isothermal calorimeter (TAM Air, TA Instruments Inc., New Castle, DE, USA) at 25 °C within 72 h, with a temperature fluctuation of less than ±0.02 °C.

## 3. Results and Discussion

### 3.1. Characteristics of Hydration Heat Evolution

**Figure 5:**
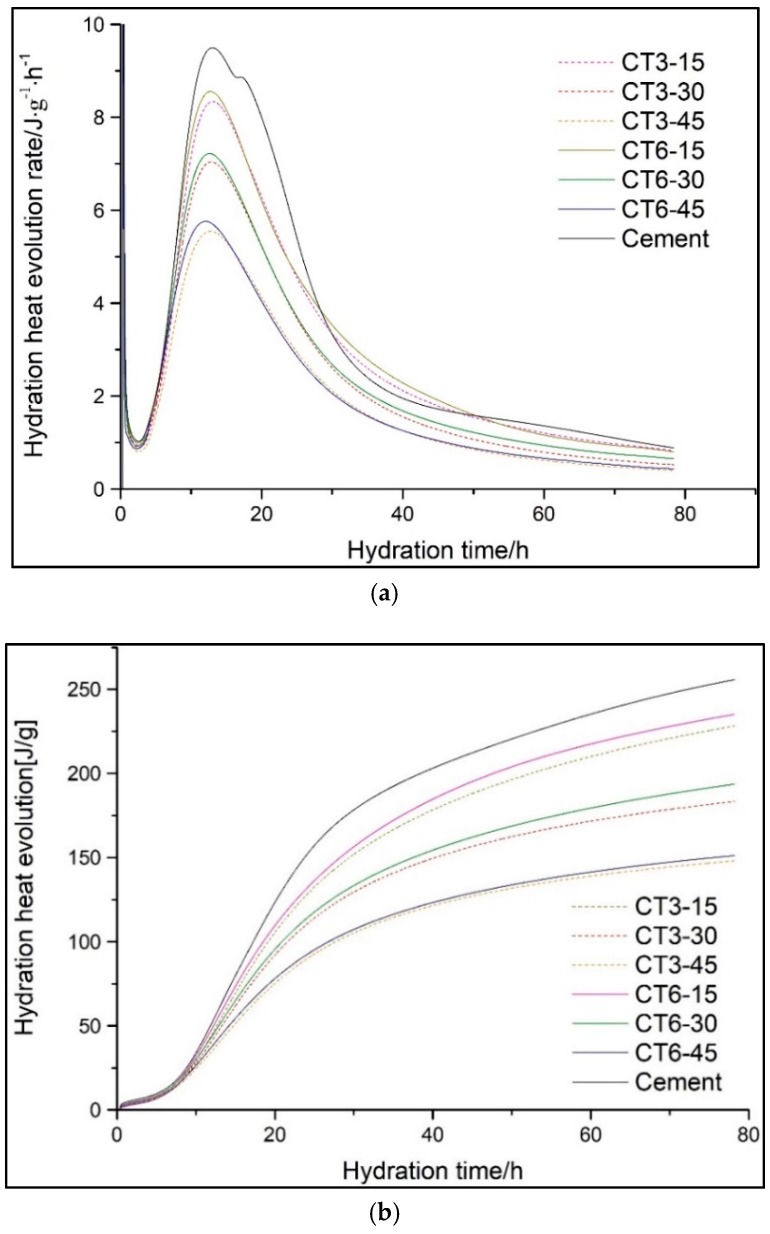
(**a**) Hydration heat evolution rate and (**b**) total hydration heat release of sample CTs.

**Figure 6:**
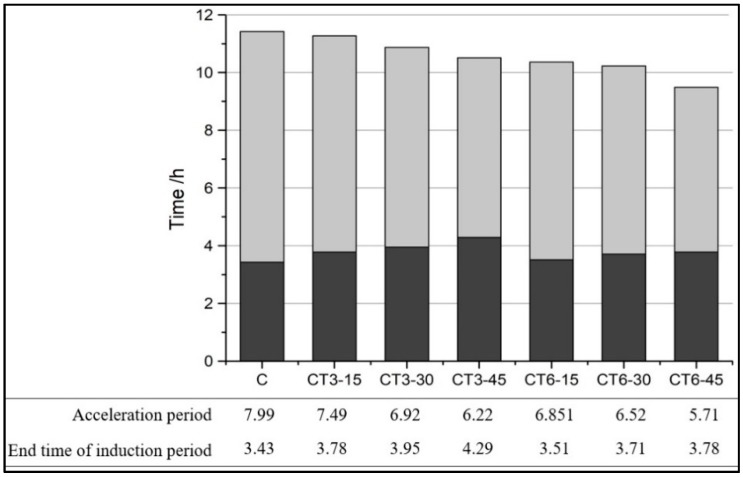
The acceleration period and end time of induction period.

Figure 5 shows the hydration heat evolution rate and total hydration heat of a cement-CT composite system (CTs) within 72 h. The acceleration period and the time at which the induction period of CTs ended are shown in Figure 6. The rate of the second exothermic peak and the total hydration heat evolution at different hydration times determined from heat evolution curves are listed in Table 4.

As shown in Figure 5a, during the first few minutes after mixing the binder with water, a sharp exothermic peak corresponding to the first peak occurs in the curves and is attributed to the quick dissolution of cement and the quick formation of ettringite [15]. Then, the first peak declines dramatically and goes into the induction period. Figure 6 indicates that there is little difference among all the samples during the induction period. The duration of the induction of cement lasts for 1.94 h and ends at about 3.43 h. With the increase in CT content, the duration of the induction period is prolonged from 2.21 to 3.1 h. It may be caused because the cement content of the cementitious system decreases with the increase of CT content. Therefore, the dissolved Ca^2+^ concentration reduces and the time when Ca^2+^ reaches supersaturation extends, which leads to the ending time of the induction period being slightly prolonged [16,17,18].

After the acceleration period, comes the strong hydration reaction of C_3_S and fast formation of C-S-H and Ca(OH)_2_ [19]. Owing to the sufficient reactant and water supply, the reaction during the acceleration period proceeds very quickly to form hydration products around the unreacted particles, which in turn postpones the hydration reaction. When the accelerating effect is equal to the delaying effect, the maximum value of the second exothermic peak is achieved. As a whole, the peak value improves and the duration time of the induction period is prolonged with the increase of CT content. In addition, the time at which the second exothermic peak is observed for all samples is slightly different and the hydration release curves tend to narrow with the increase in CT content, which corresponds to a low total hydration heat evolution. The low hydration activity and hydration degree of CT in the early stage, which mainly plays the filling role, is negligible and leads to the decrease in the overall quantity of reactants. Then, the reaction goes into the deceleration period and stable period, and the hydration reactions are much more steady and controlled by diffusion.

Moreover, it is interesting that the heat release per g of cement of the cement-CT binder clearly improves with the increase of CT content and strengthens slightly with the increase of CT fineness, as shown in Table 4. The actual water–cement ratio increases with the increase of the CT content, thus there is more water involved in the cement hydration, and the hydration rate of the cement at an early stage is accelerated even though the total hydration rate is delayed. CT particles, especially for the fine particles, can act as nucleation sites for cement hydration and accelerate its hydration. Similar results have been found for other mineral admixtures in our previous studies [20,21] time taken to observe the second exothermic peak decreases with the increase of the specific surface area. It indicates that the high CT fineness can accelerate the early hydration of composite cementitious materials.

Figure 5b shows that the total hydration heat evolution of a composite system containing CT is evidently lower than that of pure cement. Moreover, the total hydration heat evolution reduces with the increase of CT content but improves with the increase of CT fineness. As shown in Table 4, the heat emission at 72 h decreases by at least 8%, from 249.72 to 229.75 J/g, after incorporating 15% CT and decreases no less than 33%, from 222.73 to 148.30 J/g, when the CT content increases from 15 to 45%.

**Figure 7:**
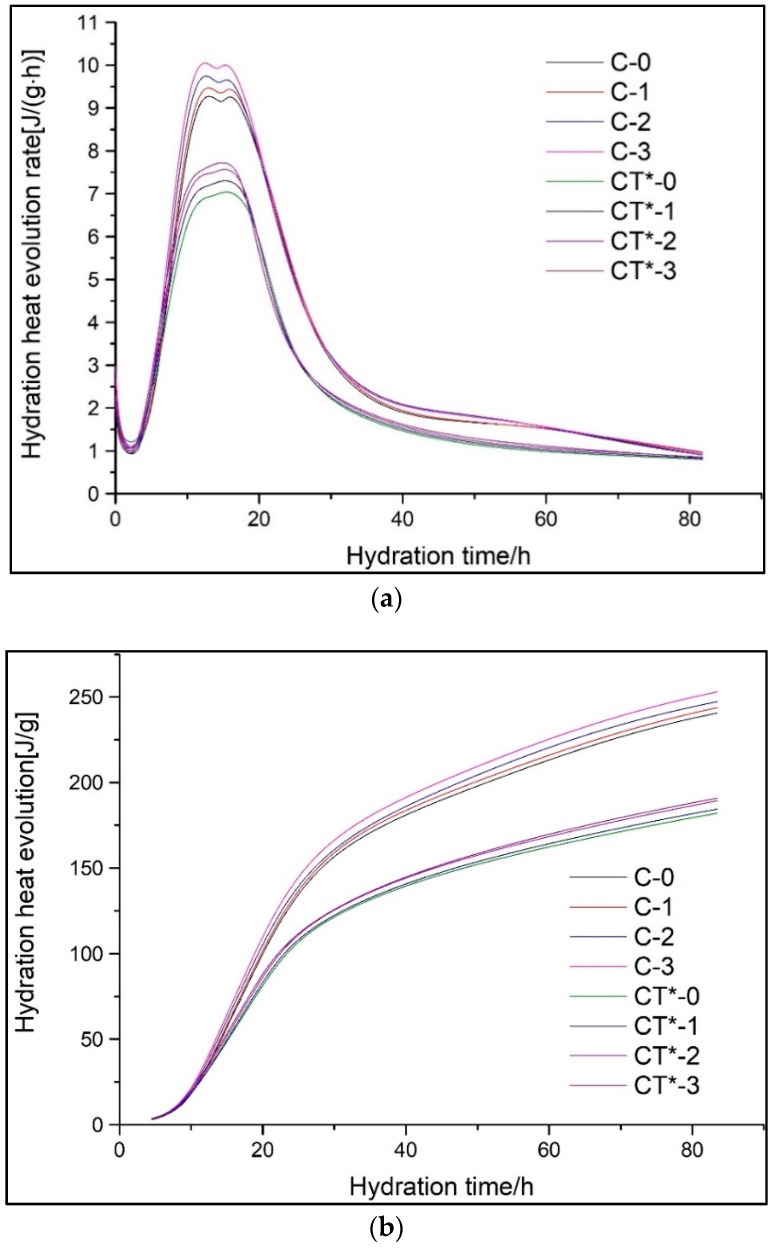
(**a**) Hydration heat evolution rate and (**b**) total hydration heat release of samples CGs and GCTs.

Figure 7 displays the hydration heat evolution rate and total hydration heat release of a cement-GO-CT composite system (GCTs). The second exothermic peak is slightly quickened as well as the value increases slightly with the GO content increasing from 0.01 to 0.03% in both pure cement and cement-CT composite system containing 30% CT. GO lamellar can serve as nucleation sites for C-S-H to nucleate and crystalize, which promotes the hydration of the cementitious system. As shown in Figure 7b, the total hydration heat also increases slightly with the increase of GO content.

### 3.2. Hydration Process Simulation

#### 3.2.1. Hydration Kinetic Model

A hydration kinetic model is often used to analyze the influence of various factors on the reaction rate and reaction direction during the hydration process, in order to reveal its control mechanism [22]. The Krstulovic–Dabic model assumes that three basic processes take place during the early hydration of the cement-based materials, namely nucleation and crystal growth (NG), phase boundary reaction (I), and diffusion (D) [23]. The three control processes may occur simultaneously, and can also occur alone or in pairs, but the hydration rate of the overall process depends on the one which reacts slowest. That is, the slowest reaction controls the reaction rate and mechanism at an early stage [24]. The Krstulovic–Dabic model also gives the basic kinetic equations to describe the three dominating processes of these three control processes as follows:

(1) $$\mathrm{NG}:\;{\left[-\ln\left(1-\alpha \right)\right]}^{1/n}=K_{1}\left(t-t_{0}\right)=K_{1}^{\prime }\left(t-t_{0}\right)$$

(2) $$I:\;{\left[1-{\left(1-\alpha \right)}^{1/3}\right]}^{1}=K_{2}R^{-1}\left(t-t_{0}\right)=K_{2}^{\prime }\left(t-t_{0}\right)$$

(3) $$D:\;{\left[1-{\left(1-\alpha \right)}^{1/3}\right]}^{2}=K_{3}R^{-1}\left(t-t_{0}\right)=K_{3}^{\prime }\left(t-t_{0}\right)$$

where *α* is hydration degree; $K_{1}\left(K_{1}^{\prime }\right)$, $K_{2}\left(K_{2}^{\prime }\right)$, $K_{3}\left(K_{3}^{\prime }\right)$ is the reaction rate constant corresponding to the hydration processes NG, I, and D; *t* is hydration time; *t*_0_ is the time when the induction period ends; *R* is the ideal gas constant; *n* is the crystal growth index that reflects the geometrical crystal growth, 1 ≤ *n* ≤ 2 [25].

When *α* is differentiated with respect to *t* in the equations above, the hydration rate of each process is obtained as follows:

(4) $$\mathrm{NG}:\;d\alpha /dt=F_{1}\left(\alpha \right)=K_{1}^{\prime }\left(1-\alpha \right){\left[-\ln\left(1-\alpha \right)\right]}^{\frac{n-1}{n}}$$

(5) $$I:\;d\alpha /dt=F_{2}\left(\alpha \right)=3K_{2}^{\prime }{\left(1-\alpha \right)}^{2/3}$$

(6) $$D:\;d\alpha /dt=F_{3}\left(\alpha \right)=3[K_{3}^{\prime }{\left(1-\alpha \right)}^{\frac{2}{3}}]/2[1-{\left(1-\alpha \right)}^{\frac{1}{3}}]$$

where $F_{1}\left(\alpha \right)$, $F_{2}\left(\alpha \right)$, and $F_{3}\left(\alpha \right)$ represent the hydration processes NG, I, and D, respectively.

Based on the total hydration emission Q(t) and the rate of hydration evolution d*Q*/d*t* obtained by isothermal conduction calorimetry, the hydration degree *α* and hydration rate d*α*/d*t* required for the kinetic simulation of hydration process are determined by the following equations [26]:

(7) $$\alpha \left(t\right)=\frac{Q\left(t\right)}{Q_{\max}}$$

(8) $$\frac{d\alpha }{dt}=\frac{dQ}{dt}\cdot \frac{1}{Q_{\max}}$$

(9) $$\frac{1}{Q}=\frac{1}{Q_{\max}}+\frac{t_{50}}{Q_{\max}\cdot t}$$

where this newly defined *Q*(*t*) is the heat released from the end of the induction period. Due to the fact that the dissolution progress is so fast that it is not always possible to be detected. Meanwhile, the induction period is also thought to make a small contribution to the total heat. Therefore, this simulation is conducted from the beginning of the second peak, namely the ending of the induction period [27]; *Q*_max_ is the total hydration heat when the reaction has completely finished and is obtained by using the Knudsen extrapolation Equation (9) to linearly fit the hydration heat evolution curves [28]: where *t*_50_ is the time required for half of *Q*_max_.

#### 3.2.2. Hydration Process Simulation of CTs

**Figure 8:**
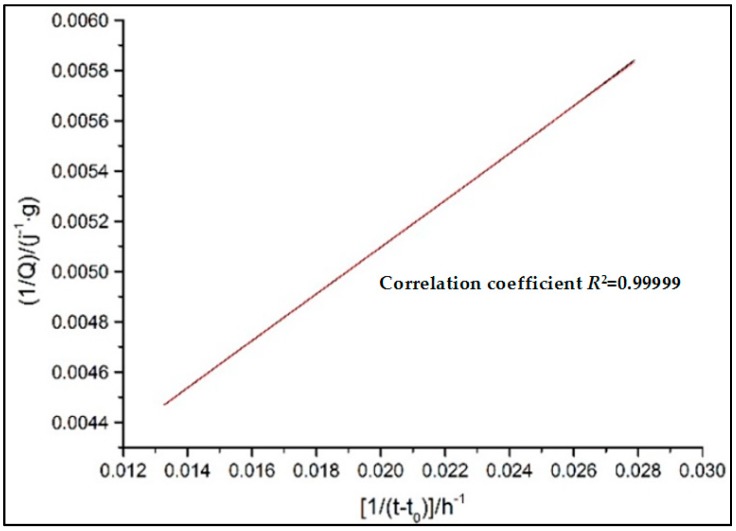
Determination of maximum hydration emission heat *Q*_max_ from linear regression.

**Figure 9:**
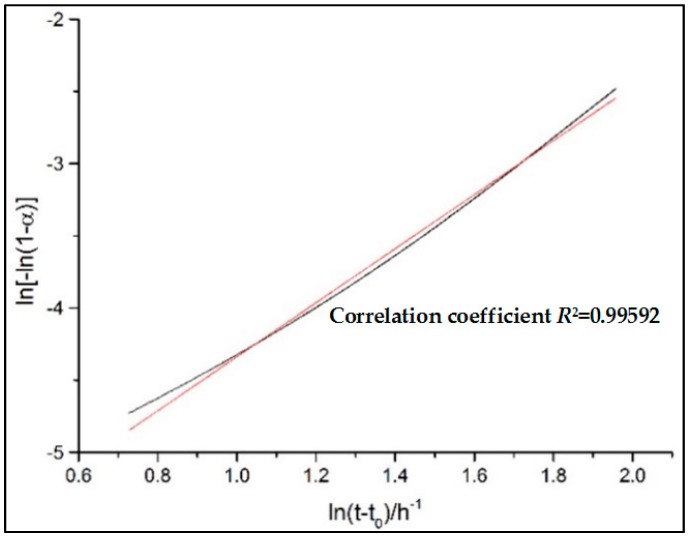
Determination of kinetic factors (*n* and $K_{1}^{\prime }$) of nucleation and crystal growth (NG) progress from linear regression.

**Figure 10:**
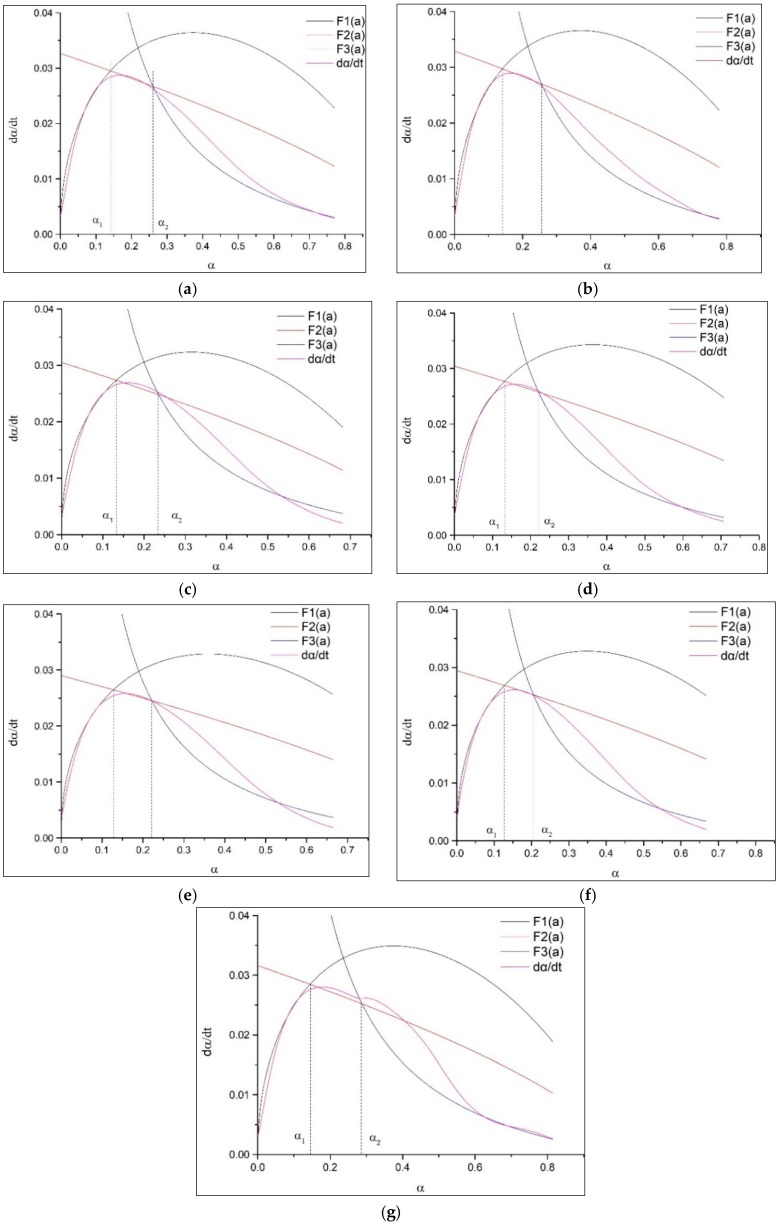
Hydration rate curves for samples CTs: (**a**) CT3-15; (**b**) CT6-15; (**c**) CT3-30; (**d**) CT6-30; (**e**) CT3-45; (**f**) CT6-45; (**g**) Cement.

In order to obtain the hydration kinetic equations of CTs, the *Q*_max_ and hydration degree *α*(*t*) is determined by Equations (7) and (9), respectively and successively at first, as shown in Figure 8. Then, the kinetic parameters $K_{1}^{\prime }$ and *n* during the nucleation and crystal growth (NG) process could be calculated by substituting *α*(*t*) into Equation (1) and linearly fitting the double logarithmic curve of $\ln\left[-\ln\left(1-\alpha \right)\right]\;\mathrm{vs}.\;\ln\left(t-t_{0}\right)$, as shown in Figure 9. $K_{2}^{\prime }$ for I process and $K_{3}^{\prime }$ for the D process can also be derived by plugging *α*(*t*) into Equations (2) and (3) and fitting the double logarithmic curve $\ln{\left[1-\left(1-\alpha \right)\right]}^{1/3}\;\mathrm{vs}.\;\ln\left(t-t_{0}\right)$ linearly. Finally, the hydration kinetic expressions, $F_{1}\left(\alpha \right)$, $F_{2}\left(\alpha \right),$ and $F_{3}\left(\alpha \right),$ characterizing the hydration rate of the NG, I, and D processes as a function of the hydration degree *α*, are acquired. The relationships of $F_{1}\left(\alpha \right)$, $F_{2}\left(\alpha \right)$, $F_{3}\left(\alpha \right)$ and dα/dt with α are also shown in Figure 10. The intersection point $\alpha_{1}$ is the turning point from NG to I, and $\alpha_{2}$ is the turning points from I to D.

From Figure 10, it can be seen that the practical hydration curves of dα/dt can be segmentally simulated by the theoretical curves, i.e., $F_{1}\left(\alpha \right)$, $F_{2}\left(\alpha \right)$, and $F_{3}\left(\alpha \right)$. It means that the hydration kinetic model could basically simulate the hydration process of all samples and the hydration undergoes three processes, namely NG, I, and D, in order. The hydration of the composite system is controlled by multiple reaction mechanisms instead of a single one.

Cement reacts with water primarily, and Ca(OH)_2_ becomes supersaturated in a few minutes and then the hydrates grow from a fixed number of nuclei [29]. For the cement–CT binder, CT particles can also serve as nucleation sites for hydrates in addition to cement grains. After that, the hydration products grow rapidly on the limited number of nuclei [30,31]. As a result, the NG process dominates the hydration process. Owing to the continued replenishment of Ca^2+^ dissolved from unhydrated particles to supply the hydration reaction, the supersaturation state of Ca^2+^ remains constant. The reaction mainly occurs at the boundary between the solid hydrates and the liquids. At that time, the phase-boundary-controlled I process plays the leading role [32]. As the hydration process continues, the hydration products increase. In the meantime, a large amount of water is consumed. The ability of water and ions to reach the surface of the unhydrated particles through the hydrated layer also becomes difficult. Therefore, the diffusion process, D, becomes the dominant process.

The kinetic parameters of the hydration process of sample CTs are listed in Table 5. The value of reaction order *n* reduces with the increase of CT dosage while improving with the increase of CT fineness, which indicates that high fineness may affect the crystal growth geometry. The rate of chemical reaction during the NG process is very fast. The rate of the NG process is about 4–5 times the rate of the I process, and about 20 times the rate of the D process. The hydration reaction during the NG process is an autocatalytic reaction. The continuous growth of the crystal nucleus leads to the increase of their boundaries, which in turn accelerates the hydration reaction of the NG process. Therefore, the hydration reaction during the NG process is fairly fast. However, the hydration reaction during the I process is mainly controlled by the ion concentration, crystal area, and growth space for hydrates. In comparison with the NG process, a lot of reactants and water are consumed and a large amount of hydration products are formed during the I process, leading to the ion concentration to decline and the growth space for hydration products to narrow. Thus, the hydration rate of the I process is much lower than that of the NG process. With the development of hydration, the hydration reaction proceeds to the D process. During this process, massive dense C-S-H is formed due to the hydration of cement and the pozzolanic reaction of CT, which wraps on the surface of unreacted particles and makes the ion immobility difficult. As a result, the hydration rate during the D process is even lower than that during the I process.

As shown in Table 5, $k_{1}^{\prime }$ decreases with the increase of CT dosage, implying that CT affects the nucleation and growth of hydrates. During the NG process, the reaction amount of CT is usually considered negligible because of its low pozzolanic activity [32]. With the increase of CT content, the cement dosage decreases, leading to the decrease of the pH value and the solubility of amorphous silicon as well as the growth rate of the crystal nucleus. Therefore, the growth rate during the NG process reduces with the increase of CT content. For the I process, the trend of the hydration rate, $k_{2}^{\prime }$, is consistent with that of $k_{1}^{\prime }$. The reaction rate of CT is much lower than that of cement. The amount of CT accounts for a large proportion of the increase in CT content, which leads to the reduction of the $k_{2}^{\prime }$ value. For the D process, a similar trend of $k_{3}^{\prime }$ is observed. A large amount of hydration products have been formed and the reaction becomes stable at this stage. On the one hand, the higher the content of cement is, the more intense the preceding hydration reaction and the denser the hydration products will be; therefore, the more difficult the ion mobility will be. Thus, the hydration reaction of the D process is weakened. On the other hand, a higher content of cement will greatly strengthen the overall hydration reaction of the cementitious system. When the positive effect leading to the improvement of the cement dosage and decrease of the CT content outweighs the negative, the whole reaction rate during the D process will decrease with the incorporation of CT.

It is also noted that the hydration duration for both the NG process and I process is shortened with the increase of CT content, indicating that the hydration reaction of the composite binder containing CT transforms from the NG to I process and from the I to D process at a lower hydration degree with the increase of CT content. The replacement of cement by CT increases the effective water to cement ratio and provides more space for hydration products during the early hydration stage. Meanwhile, CT with high fineness can also act as nucleation sites for hydration products during the NG process [19]. Therefore, the controlling effect of the NG and I process is strengthened, which leads to a sharp exothermic rate and narrow hydration duration.

#### 3.2.3. Hydration Process Simulation of Composite Cementitious System Containing GO

**Figure 11:**
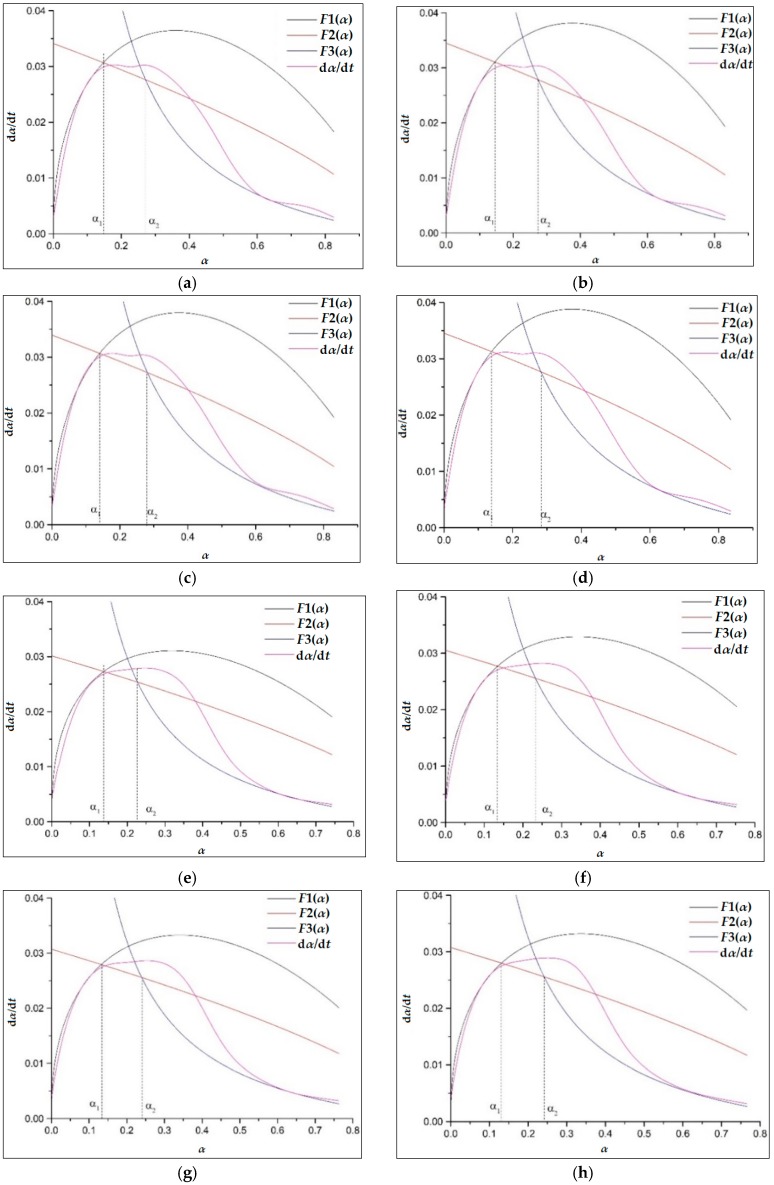
Hydration rate curves for samples of CGs and GCTs: (**a**) C-0; (**b**)C-1; (**c**)C-2; (**d**) C-3; (**e**) CT*-0; (**f**) CT*-1; (**g**) CT*-2; (**h**) CT*-3.

Figure 11 shows the simulated and practical hydration exothermic curves of composite cementitious materials containing GO and CT. It is observed that curves, $F_{1}\left(1\right),\;F_{2}\left(2\right)$, and $F_{3}\left(3\right)$, simulate the experimental hydration curves well, which validates that the hydration of both the cement-GO system (CGs) and cement-GO-CT system (GCTs) has a complicated process with a multiple reaction mechanism. In particular, the hydration process of CGs and GCTs after the induction period are the NG, I, and D processes in turn. Moreover, the hydration mechanism of the composite systems with GO is similar to that without it (C-0, CT*-0).

The kinetic parameters of the hydration process of composite cementitious materials are given in Table 6. The duration of the NG process is shortened while the I process is prolonged. For the CG system, the value of $\alpha_{1}$ decreases from 0.1483 to 0.1377 while $\alpha_{2}-\alpha_{1}$. increases from 0.2702 to 0.2833 while GO content increases from 0 to 0.03%. With regard to the GCTs system, the value of $\alpha_{1}$ decreases from 0.1375 to 0.1306, while $\alpha_{2}-\alpha_{1}$ increases from 0.2262 to 0.2413 along with the increase in the GO content from 0 to 0.03%. It illustrates that GO promotes the nucleation and crystal growth process by acting as nucleation sites, and leads to a higher reaction rate but a shorter duration of the NG process. This also be confirmed by the fact that $k_{1}^{\prime }$ increases with the GO content. However, the hydration process during the I process is prolonged with the increase of GO dosage. It may be because GO improves the overall quantity of nucleation sites for crystal growth. In this case, the boundaries between the growing crystals and the solutions may also increase, leading to a longer duration to finish the I process. It also can be observed that the values of $\alpha_{1}$ and $\alpha_{2}-\alpha_{1}$ of GCTs are even less than those of CGs. It is also confirmed that the replacement of CT leads to a short hydration duration.

Overall, based on the data in Table 5 and Table 6, the hydration rates, $k_{1}^{\prime }$, $k_{2}^{\prime }$, and $k_{3}^{\prime }$ of the three controlling processes of the composite cementitious system decrease with the increase of CT content, but improve slightly with the increase of CT fineness. Although CT exerts an adverse effect on the early hydration, this can be slightly compensated for by the increase of CT fineness and can be overturned by the incorporation of GO. GO evidently accelerates the hydration of composite materials with the fact that $k_{1}^{\prime }$, $k_{2}^{\prime }$, and $k_{3}^{\prime }$ gradually increase with GO dosage. Additionally, with the increase of CT dosage as well as its fineness, the NG and I process are gradually shortened. GO enhances the controlling effect of the NG process of the cementitious systems with or without CT, thus promoting the early hydration.

## 4. Conclusions

(1) Copper tailing powder, as a replacement for cement, reduces the early heat release rate and heat discharge of the cementitious system. The hydration rate and the total heat release improve with the decease of the content of copper tailing powder and the increase of the fineness of copper tailing powder. At the same time, graphene oxide can further improve the hydration rate and hydration heat of the cementitious system.
(2) The Krstulovic–Dabic kinetic model can be used to characterize the controlling process during the hydration. The hydration process of the composite cementitious materials containing copper tailing powder and graphene oxide is controlled by a multiple reaction mechanism, namely nucleation and crystal growth (NG), phase boundary reaction (I), and diffusion (D), in that order.
(3) The hydration rates, $k_{1}^{\prime }$, $k_{2}^{\prime }$, and $k_{3}^{\prime }$ of the three controlling processes reduce with the increase of the content of copper tailing powder, and improve with the increase of the fineness of copper tailing powder and the dosage of graphene oxide. Graphene oxide enhances the controlling effect on the nucleation and crystal growth process of the cementitious systems with or without copper tailing powder, thus promoting the early hydration.

## Figures and Tables

**Table 1:** The main chemical compositions of cement and copper tailing powder (CT)/Mass, %.

Compositions	SiO_2_	Al_2_O_3_	CaO	Fe_2_O_3_	MgO	SO_3_	TiO_2_	K_2_O	Na_2_O	P_2_O_5_	CuO
Cement	21.25	2.91	63.09	3.24	0.68	3.36	0.31	1.12	0.31	0.17	-
CT	39.15	5.49	31.76	8.39	5.37	1.21	0.21	0.64	1.32	0.11	0.08

**Table 2:** Mixture proportions I (samples cement-CT system (CTs))/Mass, %.

Sample	Grinding Time	CT	W/C
CT3-15	30 min	15%	0.4
CT3-30	30 min	30%	0.4
CT3-45	30 min	45%	0.4
CT6-15	60 min	15%	0.4
CT6-30	60 min	30%	0.4
CT6-45	60 min	45%	0.4
C	/	0%	0.4

**Table 3:** Mixture proportions II (samples cement-GO system (CGs) and samples cement-GO-CT system (GCTs))/Mass, %.

Sample		GO Content	Cement	CT	W/C
CGs	C-0	0	100%	0	0.4
	C-1	0.01%	100%	0	0.4
	C-2	0.02%	100%	0	0.4
	C-3	0.03%	100%	0	0.4
GCTs	CT*-0	0	70%	30%	0.4
	CT*-1	0.01%	70%	30%	0.4
	CT*-2	0.02%	70%	30%	0.4
	CT*-3	0.03%	70%	30%	0.4

**Note:** Taking CT6-30 in mixture proportions I for example, 6 indicates that the grinding time of CT is 60 min, 30 represents that the mixing content of CT is 30%; For CT*-2 in mixture proportions II, CT* is the simplified name of CT6-30, 2 stands for 0.02% dosage of graphene oxide (GO).

**Table 4:** Characteristic values of hydration heat evolution curves of samples at 25 °C.

Sample	Rate of the Second Heat Emission Peak *q*_max_ (J/g·h)	Total Heat Release (J/g)				Heat Release Per g of Cement (J)			
		12 h	48 h	60 h	72 h	12 h	48 h	60 h	72 h
CT3-15	8.341	44.7	193.2	210.0	222.7	52.6	227.3	247.1	262.0
CT3-30	7.044	41.0	160.2	171.6	179.9	58.6	228.9	245.1	257.0
CT3-45	5.529	34.9	130.0	139.0	145.4	63.5	236.4	252.7	264.4
CT6-15	8.541	48.2	200.6	217.5	229.8	56.7	236.0	255.9	270.4
CT6-30	7.203	43.7	166.2	179.4	189.3	62.4	237.4	256.3	270.4
CT6-45	5.768	37.6	132.0	141.4	148.3	68.4	240.0	257.1	269.6
C	9.479	51.6	217.3	235.3	249.7	51.6	217.3	235.3	249.7

**Table 5:** Kinetic parameters of the hydration process of sample CTs.

Sample	N	$k_{1}^{\prime }$	$k_{2}^{\prime }$	$k_{3}^{\prime }$	$\alpha_{1}$	$\alpha_{2}$	$\alpha_{2}-\alpha_{1}$	$Q_{max}^{\prime }$
Cement	1.8843	0.04425	0.01053	0.00226	0.1456	0.2860	0.1404	305.983
CT3-15	1.8521	0.04414	0.01088	0.00208	0.1412	0.2601	0.1189	290.475
CT3-30	1.8188	0.04260	0.01016	0.00169	0.1312	0.2340	0.1028	261.525
CT3-45	1.7522	0.04080	0.00966	0.00155	0.1280	0.2208	0.0928	215.114
CT6-15	1.8788	0.04488	0.01097	0.00206	0.1404	0.2558	0.1154	295.598
CT6-30	1.8401	0.04236	0.01014	0.00220	0.1318	0.2204	0.0886	265.947
CT6-45	1.7988	0.04136	0.00982	0.00145	0.1280	0.2055	0.0775	220.486

**Table 6:** Kinetic parameters of hydration process of sample CGs and CTGs.

Sample		N	$k_{1}^{\prime }$	$k_{2}^{\prime }$	$k_{3}^{\prime }$	$\alpha_{1}$	$\alpha_{2}$	$\alpha_{2}-\alpha_{1}$	$Q_{max}^{\prime }$
CGs	C-0	1.806	0.04520	0.01137	0.00228	0.1483	0.2702	0.1219	306.112
	C-1	1.878	0.04599	0.01149	0.00233	0.1456	0.2742	0.1286	309.310
	C-2	1.881	0.04600	0.01151	0.00236	0.1404	0.2794	0.1390	312.495
	C-3	1.894	0.04705	0.01158	0.00241	0.1377	0.2833	0.1456	314.794
GCTs	CT*-0	1.621	0.04060	0.01005	0.00165	0.1375	0.2262	0.0887	251.889
	CT*-1	1.700	0.04182	0.01017	0.00172	0.1341	0.2333	0.0992	256.410
	CT*-2	1.717	0.04235	0.01023	0.00179	0.1318	0.2401	0.1083	263.850
	CT*-3	1.725	0.04251	0.01025	0.00181	0.1306	0.2413	0.1107	267.380
